# Botulinum Toxin Type A as a Therapeutic Agent against Headache and Related Disorders

**DOI:** 10.3390/toxins7093818

**Published:** 2015-09-23

**Authors:** Siro Luvisetto, Parisa Gazerani, Carlo Cianchetti, Flaminia Pavone

**Affiliations:** 1 National Research Council (CNR) of Italy, Institute of Cell Biology and Neurobiology, Roma 00185, Italy; E-Mail: flaminia.pavone@cnr.it; 2 Center for Sensory-Motor Interaction, Department of Health Science and Technology, Faculty of Medicine, Aalborg University, Aalborg East 9100, Denmark; E-Mail: gazerani@hst.aau.dk; 3 Former Professor of Child & Adolescent Neuropsychiatry, University of Cagliari, Cagliari 09124, Italy; E-Mail: cianchet@unica.it

**Keywords:** botulinum toxin, headache, migraine, tension-type headache, cluster headache, cephalalgias, animal pain model, human pain model, clinical trials

## Abstract

Botulinum neurotoxin A (BoNT/A) is a toxin produced by the naturally-occurring *Clostridium botulinum* that causes botulism. The potential of BoNT/A as a useful medical intervention was discovered by scientists developing a vaccine to protect against botulism. They found that, when injected into a muscle, BoNT/A causes a flaccid paralysis. Following this discovery, BoNT/A has been used for many years in the treatment of conditions of pathological muscle hyperactivity, like dystonias and spasticities. In parallel, the toxin has become a “glamour” drug due to its power to ward off facial wrinkles, particularly frontal, due to the activity of the mimic muscles. After the discovery that the drug also appeared to have a preventive effect on headache, scientists spent many efforts to study the potentially-therapeutic action of BoNT/A against pain. BoNT/A is effective at reducing pain in a number of disease states, including cervical dystonia, neuropathic pain, lower back pain, spasticity, myofascial pain and bladder pain. In 2010, regulatory approval for the treatment of chronic migraine with BoNT/A was given, notwithstanding the fact that the mechanism of action is still not completely elucidated. In the present review, we summarize experimental evidence that may help to clarify the mechanisms of action of BoNT/A in relation to the alleviation of headache pain, with particular emphasis on preclinical studies, both in animals and humans. Moreover, we summarize the latest clinical trials that show evidence on headache conditions that may obtain benefits from therapy with BoNT/A.

## 1. Botulinum Neurotoxins: Biological Properties and Mechanism of Action

Botulinum neurotoxins (BoNTs) are produced as multimolecular complexes by anaerobic bacteria of the genus *Clostridium* [1,2,3]. Seven different serotypes of BoNTs have been characterized (A–G), and these serotypes are active on many different types of vertebrates [2]. Recently, a new serotype (BoNT/H) has been proposed [4], but it still remains to be experimentally validated.

BoNTs are proteins of about 1300 amino acids and consist of three domains of a similar size (about 50 kDa) [2,5,6]. The NH_2_-terminal domain, which is named the *L*-chain domain, is a Zn^2+^-endopeptidase that represents the catalytic domain expressing the protease activity. The other two domains, which are covalently bound to form the *H*-chain, are the central domain, responsible for the membrane translocation of the *L*-chain into the neuronal cytosol, and the COOH-terminal domain, which consists of two equally-sized subdomains, responsible for the neurospecific binding.

The cellular action of BoNTs occurs as a four-step mechanism [2,7]: (i) binding of BoNTs on the neuronal presynaptic membrane, via interaction with gangliosides, synaptic vesicle protein 2 (SV2) and/or synaptotagmin, depending on the serotype; (ii) internalization of BoNTs, via endocytosis of the BoNTs-receptor complex inside the neurons; (iii) translocation of BoNTs’ *L*-chain from the endocytosed vesicle to the neuronal cytosol; and, finally, (iv) cleavage by Zn^2+^-endopeptidase activity of specific proteins involved in neuroexocytosis [5,8]. These proteins are: SNAP-25 cleaved by BoNT/A, /E and /C; VAMP/synaptobrevin cleaved by BoNT/B, /D, /F and /G; and syntaxin cleaved by BoNT/C. All of these proteins are involved in the assembly of the SNARE (soluble *N*-ethylmaleimide-sensitive factor attachment protein receptors) proteins’ core complex, which is fundamental for correct docking and fusion of neurotransmitter vesicle with neuronal membranes [9,10]. The cleavage of one of these proteins is sufficient to prevent the correct assembly of the SNARE core complex and the consequent fusion of synaptic vesicles with the neuronal presynaptic membrane, thus inhibiting neurotransmitter release [5,11]. This effect is reversible, being the duration of the action dependent on the serotype. Further details of the mechanism of binding, internalization and mode of action of the different BoNT serotypes is out of the scope of this review and may be properly found elsewhere.

## 2. BoNT/A in Medicine: Brief History

The medical use of BoNTs as therapeutic drugs began after purification of BoNT/A into crystalline form [12,13] and the subsequent discovery that the injection of small amounts of BoNT/A into a hyperactive muscle blocked the release of acetylcholine (ACh) from motor nerve endings, causing temporary “muscle relaxation” [14]. This finding led to the first use of BoNT/A as a therapeutic drug to treat human strabismus as an alternative to the conventional surgery [15]. After these pioneering studies, therapeutic uses of BoNT/A extended to a wide variety of neurological disorders originating from the hyperfunctionality of cholinergic terminals, such as the spasmodic torticollis, blepharospams, facial emispams, dystonia and spasticity [16,17].

However, over the years, it has been recognized that BoNTs cannot be considered exclusively “cholinergic” toxins. In fact, despite the fact that they act preferentially on nerve terminals between motoneurons and muscle fibers, BoNTs can block the neural transmission from other synapses, cholinergic or not [18], and many preclinical studies demonstrated that BoNTs (mainly BoNT/A) block the Ca^2+^-evoked neuroexocytosis of neurotransmitters other than ACh [19], including those involved in pain transmission. Nowadays, the clinical indications for BoNT/A are rapidly growing, ranging from treatment of overactive skeletal and smooth muscles, to management of hypersecretory (hyperhidrosis, sialorrhea) and painful disorders, such as myofascial pain syndrome, trigeminal neuralgia and chronic migraine [20,21,22]. The potential for BoNTs as a treatment for headaches was discovered casually during clinical trials to determine the efficacy of BoNT/A as a treatment of cranio-facial dystonia: patients reported a reduction of headache attacks together with beneficial effects of BoNT/A on dystonia [23]. A retrospective review of headache patients who were receiving BoNT/A injections for neurology, otolaryngology or cosmetic indications also suggested a reduced headache frequency [24]. This led to the first prospective, non-randomized, open-label study of 106 patients designed to determine a relationship between BoNT/A treatment and the reduction of headache [25].

In this review, we first summarize preclinical studies, conducted both in animals and humans, relevant to the understanding of the mechanisms of action of BoNT/A in relation to its use in the treatment of headaches, and subsequently, we summarize clinical studies in which BoNT/A has been used as a therapy against headaches. Since BoNT/A is the serotype most commonly used in clinical practice, we have only focused on the studies that used this serotype. Although BoNT/A is marketed under different names, we prefer to maintain the acronym BoNT/A to indicate serotype A of botulinum neurotoxin. Nevertheless, in the sections devoted to research on humans, the trade name of the toxin will be properly specified in both experimental and clinical trials.

## 3. Headache: Definitions and Classification

Before reviewing available evidence in favor or against the use of BoNT/A for the treatment of headaches, we briefly illustrate the current knowledge of the different types of headaches. Headache can be originated from a series of causes, e.g., intracranial tumors, head trauma, vascular and inflammatory disorders. Pain is mediated by sensitive structures that are located both extracranial (sinus; eyes/orbits; ears; teeth; temporomandibular joint; blood vessels) and/or intracranial (arteries of the circle of Willis; dural venous sinuses; veins; meninges).

Based on the indications of the Headache Classification Committee of the International Headache Society, reported in the third edition of The International Classification of Headache Disorders [26], headaches are broadly classified as “primary”, or idiopathic headaches (headache is itself the disease), and “secondary”, or symptomatic headaches (headache is only a symptom of another underlying disease). Primary headaches are grouped into four main subgroups: migraine, tension-type headache (TTH), cluster headache and trigeminal autonomic cephalalgias (TACs), as well as other primary headaches. Secondary headaches represent a vast variety of headache and are classified depending on the etiology. Thus, there are secondary headaches originating from disorders of extracranial structures (e.g., sinusitis, otitis, glaucoma, temporomandibular joint dysfunction, *etc*.) or of intracranial structures (e.g., vasculitis, venous thrombosis, tumors, abscesses, meningitis, *etc*.). Headaches may be also secondary to disorders of metabolism (e.g., hypothyroidism), of homeostasis (e.g., hypoxia, hypercapnia) and to the exposure to or use of substances (e.g., nitrous oxide, alcohol). In view of the vastness of the subject and considering that secondary headaches have a complex origin and different therapeutic targets, in this review, we focus only on primary headaches (limited to migraine, TTH, TCAs; see the summary in Table 1) and the cranial (trigeminal and occipital) neuralgias.

**Table 1:** Primary headaches.

Headaches	Migraine	Tension-Type Headache (TTH)	Trigeminal Autonomic Cephalalgias (TACs)
**Subtypes**	- Migraine without aura - Migraine with aura - Hemiplegic migraine - Chronic migraine	- Infrequent episodic TTH - Frequent episodic TTH - Chronic TTH	- Cluster headache (CH) (episodic or chronic) - Paroxysmal hemicrania (episodic or chronic) - Short-lasting unilateral neuralgiform headache (episodic or chronic) - Hemicrania continua
**Pain**	- Throbbing - Moderate to severe	- Pressing/tightening - Mild to moderate	CH: extremely severe
**Associated Symptoms**	- Nausea - Vomiting - Photophobia - Phonophobia	- None	CH: -conjunctival injection/tearing - rhinorrhea - sweating - ptosis - miosis
**Location**	- Most frequently unilateral (hemicranial)	- Bilateral	- Strictly unilateral - Mainly temporal-orbital
**Duration Frequency**	- 4/72 h	- 30 min to 7 days	CH: occurring in periods with several attacks each day, each 15′ to 3-h duration
**Sex Ratio**	F > M *	F > M *	M > F *
**Possible Triggers**	- Hormonal changes - Stress	- Stress	- Alcohol - Nitroglycerine

* F = female; M = male.

The pathophysiology of primary headaches is very complex, and the mechanisms that cause primary headaches are not completely known. There have been different theories over time that attempt to explain what is the cause of these headaches. Headaches may be caused by the activation of sensory nerves that release peptides causing inflammation in arteries, dura and meninges and also vasodilation. Concerning migraine, the exact mechanism inducing head pain is likely to be multifactorial and to involve more than one level of the nervous system. Some evidence supports a primary role of some brain structures: migraine is thought be caused by brainstem neuronal hyperexcitability, cortical spreading depression (CSD), abnormal release of neurotransmitters/neuropeptides and trigeminal system activation [27]. Other data support the role of peripheral activation via the sensory nerves surrounding blood vessels of the head and neck [28]. The potential candidate vessels include dural arteries, pial arteries and extracranial arteries, such as those of the scalp. The role of vasodilatation of the extracranial arteries in particular is also believed to be crucial [29,30].

About the other primary headaches, TTH is thought to be caused by the activation of peripheral nerves in the head and neck muscles [31], while cluster headaches (CH) and TACs involve overactivation of the trigeminal nerves and hypothalamus, but the precise cause is still unknown [32]. Trigeminal and occipital neuralgias are characterized by accesses of neuralgic pain in the sensory distribution of the trigeminal (one or more of its braches) and great occipital nerves; trigeminal neuralgias differ from the TCAs in several respects and particularly for the absence of accompanying autonomic symptoms.

## 4. BoNT/A and Headache: Is BoNT/A Effective at Treating Pain from Headaches?

The analgesic effect of BoNT/A has generally been attributed to muscular relaxation. This notwithstanding, there were reports in the literature stating that patients experience pain relief shortly after BoNT/A treatment [33,34], *i.e.*, before any muscle-relaxing action of the toxin, or that the pain relief is still maintained after muscle power returned to normal [35]. In such cases, the pain relief cannot be ascribed to abolition of muscle hyperactivity. This suggests that the analgesia produced by BoNT/A may be associated with more complex mechanisms than the simple muscular relaxation. In this section, we will discuss mechanism-based evidence for the antinociceptive effects of BoNT/A, as well as derived from animal (Section 4.1) and experimental human models (Section 4.2).

### 4.1. Mechanism-Based Evidence for the Analgesic Actions of BoNT/A: In Vitro and In Vivo Animal Studies

Nociceptive sensory endings release calcitonin gene-related peptide (CGRP) and substance P (SP) in response to noxious stimuli. CGRP modulates the cholinergic system, facilitates glutamatergic transmission and induces vasodilation on arterial smooth muscle. SP acts on mast cells to induce the release of histamine and cytokines, which directly sensitize or excite nociceptors. Like CGRP, SP is also a potent vasodilator. Important support for CGRP as a key player in the trigeminal system came from the findings that CGRP-containing neurons are most frequent in the human trigeminal ganglion [36,37] and that cortical spreading depression (CSD), a causal effector of migraine [27], triggers immediate release of neuropeptides [38,39,40]. Consistently, increased CGRP and SP contents were found in potassium-induced CSD in the trigeminal nociceptive system in rats [41]. Based on this evidence and considering its ability to inhibit neuroexocytosis, BoNT/A seems to be an ideal candidate to contrast pain associated with headache disorders.

Pioneering *in vitro* studies show that BoNT/A blocks the Ca^2+^-dependent release of SP from embryonic rat dorsal root ganglia neurons [42,43] or the K^+^(or bradykinin)-dependent release of CGRP from cultured neurons derived from rat trigeminal ganglia [44,45,46]. The ability of BoNTs to inhibit the release of CGRP and SP has been proven also for other botulinum neurotoxin serotypes, such as BoNT/D [47]. The effect of BoNT/A on immunoreactive levels of CGRP and SP was investigated in a model of migraine induced by nitroglycerine (NTG) in rats [48]. The authors found that local injection of BoNT/A, s.c. administered into the frontal and temporal area two hours after NTG administration, suppressed NTG-induced release of CGRP and SP in jugular plasma samples and in medulla oblongata. Finally, the injection of BoNT/A into craniofacial muscles of the rat could decrease the mechanical sensitivity of temporalis muscle nociceptors through inhibition of glutamate release and attenuation of provoked release of CGRP and SP from muscle nociceptors [49].

Other studies demonstrate that BoNT/A inhibits CGRP release from trigeminal ganglion neurons and eliminates the excitatory effects of CGRP in brain stem sensory neurons sensitized by capsaicin, a potent activator of the transient receptor potential (TRP) vanilloid receptor type 1 (TRPV1) [46,50]. TRPV1 is a non-selective ligand-gated cation channel, preferentially expressed in small sensory neurons [51], that responds to noxious heat, protons and chemicals, such as capsaicin. It plays a critical role in pain and neurogenic inflammation associated with tissue injury, inflammation and nerve lesions. As a result of its ability to interact with SNARE-dependent trafficking of TRPV1 [52], BoNT/A might reduce pain and neurogenic inflammation induced by capsaicin. Accordingly, a histological study on the expression of TRPV1 in the trigeminal system demonstrated that the mechanism by which BoNT/A reduces TRPV1 expression involves the inhibition of TRPV1 plasma membrane trafficking and proteasome-mediated degradation in the cytoplasm [53]. In addition to the regulation of neuronal activity, the activation of TRP channels is implicated in a variety of non-neuronal processes, including many endothelial functions, ranging from control of vascular tone and regulation of vascular permeability to angiogenesis and vascular remodelling [54]. Vasodilation of meningeal arteries may contribute to triggering migraine attacks [55]. Together with TRPV1, also the transient receptor potential ankyrin 1 (TRPA1), another non-selective cation channel belonging to the TRP channel superfamily widely expressed in neurovascular tissues [56,57], acts as a vasodilator component of neurogenic inflammation. TRPA1, normally coexpressed with TRPV1, is directly activated by compounds causing a burning sensation, such as allyl isothiocyanate (AITC), the main component of mustard oil, horseradish and wasabi [58].

Luvisetto *et al.* [59] analyzed the effect of the pretreatment with BoNT/A on pain evoked by injection of capsaicin or AITC in proximity of a vascular structure in mice. Authors demonstrated the analgesic effect of BoNT/A against pain evoked by capsaicin, agonist of TRPV1, and AITC, agonist of TRPA1. This finding is coherent with the *in vitro* demonstration of the inhibition of the expression of TRPV1 by BoNT/A [53], through an action of BoNT/A on the proteins responsible for the trafficking or translocation of these receptors, and gives support to the possible action of BoNT/A on primary headaches, in which several data indicate a role of both TRPV1 [60,61,62] and of TRPA1 [63,64,65].

In a very elegant research on rats, Burstein *et al.* [66] identified 43 C- and 36 Aδ-meningeal nociceptors and measured their spontaneous and evoked firing before and after BoNT/A administration to intracranial dura and extracranial suture-receptive fields. This study provides direct evidence for the ability of BoNT/A to inhibit mechanical nociception in peripheral trigeminovascular neurons, suggesting that BoNT/A interferes with neuronal surface expression of high threshold mechanosensitive ion channels, by preventing their fusion into the nerve terminal membrane. As outlined below, in the context of migraine, inhibition of mechanical pain signals from meningeal and other trigeminovascular nociceptors to the spinal trigeminal nucleus might be the most critical mechanism for the analgesic action of BoNT/A [66].

It has also been suggested that BoNT/A injected peripherally can reach the CNS [67]. Indirect evidence based on immunohistochemical detection of the cleaved SNAP-25 has suggested that functional BoNT/A not only reaches the central endings of nociceptor axons, but may also act on neurons in the CNS [68]. It has been suggested that BoNT/A antinociceptive action might be associated with the activity of the endogenous opioid system involving μ-opioid receptors [69,70].

Elucidating the important role of glial cells in pain [69] has opened up a new possibility for testing non-neuronal effects of BoNT/A in association with its antinociceptive/analgesic efficacy. In fact, it has been shown that satellite glial cells, in the trigeminal ganglion, contain SNAP-25 and release glutamate that is blocked by BoNT/A [71]. Similarly, cleaved SNAP-25 was found in dorsal root ganglia after peripheral injection of BoNT/A in the plantar surface of mice hind paw [72]. This finding is important since it demonstrates that BoNT/A may also reach and block vesicular release of glutamate from glial cells in the peripheral nervous system at the level of the sensory ganglia.

### 4.2. Mechanism-Based Evidence for the Analgesic Actions of BoNT/A: Human Experimental Pain Studies

Animal pain models are highly beneficial in studying pain mechanisms or the antinociceptive action of potential analgesics [73,74]; however, since pain is a complex multi-dimensional experience, animal models cannot fully mimic the complex range of clinical phenomena and, hence, only provide some information when analgesic compounds are being tested in these models [75,76]. Therefore, for better understanding of the complexity and heterogeneity of human pain and the role of various factors, such as genetic, biological and psychological parameters, in pain and analgesic responses, well-characterized human-based pain research is required. Human experimental pain models have been introduced mimicking aspects of clinical pain conditions, such as hyperalgesia and allodynia, that can assist in the evaluation of analgesic effects [77,78]. However, no ideal human model of pain exists.

Human experimental pain models are designed based on standardized stimulation (e.g., mechanical, chemical, thermal, electrical) followed by a wide range of assessments of the evoked responses (e.g., quantitative sensory tests, neuroimaging, microdialysis) in healthy human volunteers [79,80]. These models utilizing multi-modal, multi-tissue approach can bridge the missing step between animal and clinical pain research and assist in proof-of-concept studies [81].

Several human experimental pain models have been employed to investigate the potential analgesic action of BoNT/A [82,83,84,85,86,87,88,89,90], and although they yielded both negative and positive outcomes, they have provided important mechanism-based principles that can be proposed for some analgesic actions of BoNT/A [91]. These studies were designed as double-blind and placebo-controlled studies, where BoNT/A was applied into the forearm, thigh or forehead of healthy volunteers. Selected locations are either based on ethical issues, accessibility or to mimic conditions similar to a specific pain condition. The experimental pain models were diverse, including capsaicin, glutamate, electrical or ultraviolet B (UVB) irradiation models. These studies were focused on BoNT/A-based products of onabotulinum toxin A (Botox^®^, Allergan, Inc., Irvine, CA, USA) or abobotulinum toxin A (Dysport^®^, Ipsen Biopharm Limited, Wrexham, UK).

BoNT/A is proposed to prevent peripheral nerve sensitization induced by local neuromodulator release and to indirectly attenuate nervous system sensitization that could be manifested as allodynia and hyperalgesia [91]. Therefore, success or failure of a human experimental pain model to demonstrate an antinociceptive effect of BoNT/A must be discussed in terms of the chosen experimental pain model, BoNT/A dose and location of BoNT/A administration relative to the pain stimulus [91]. Below, several of these experimental pain models and the effect of BoNT/A on their manifestations are presented.

Blersch *et al.* [83] evaluated the effect of BoNT/A (Dysport^®^; 100 U) *vs.* placebo on cutaneous nociception in forearms of healthy humans. Local electrical stimulation was applied, and heat and cold pain thresholds within the treated skin areas were measured with quantitative sensory testing (QST). The tests were done before BoNT/A treatment and after four and eight weeks. Results from this study showed no direct peripheral antinociceptive effect of BoNT/A. Krämer *et al.* [87] tested the effect of intracutaneous BoNT/A (Botox^®^; 5, 10, 20 U) *vs.* saline in healthy volunteers on transcutaneous electrical stimulation-elicited pain, mechanical hyperalgesia and neurogenic flare on Days 1, 2, 3, 7 and 14 after the injection. The study revealed only a limited analgesic effect of BoNT/A (reduction of electrically-induced pain by about 10%). Hyperalgesia to pin-prick and allodynia after electrical stimulation remained unchanged. The size of electrically-induced flare was smaller in the BoNT/A-treated arm. The electrical stimulation applied in these reports does not seem to be sensitive to BoNT/A [83,87]. BoNT/A has been known to not block action potential conduction [92], so the electrical stimuli applied in these models could bypass the nociceptive nerve terminals, where the toxin is active [91,92,93]. However, if the electrical stimulation could release some neuropeptides, for example CGRP, a change in peripheral blood flow (neurogenic flare) should be expected [91]. Neurogenic flare, e.g., resulting from the release of CGRP from the peripheral nociceptive nerve terminals, is expected to be reduced or blocked by BoNT/A, as Kramer *et al.* [87] observed. These results are in line with preclinical studies where neurogenic flare mediated by CGRP was more sensitive to BoNT/A than is pain and allodynia [91]. Another mechanism-based lesson learned from the electrical models is that the custom-made concentric electrode used in the study by Blersch *et al.* [83] produced a pinprick-like pain that is most likely evoked following selective depolarization of A-δ fibers in the skin. Recently, Paterson *et al.* [94] hypothesized that BoNT/A could block nociceptor transduction, since this group observed that intradermal administration of BoNT/A in healthy volunteers produced a marked and specific decrease in noxious mechanical pain sensitivity.

The capsaicin pain model, based on excitation of the sensory neurons via binding of capsaicin to TRPV1 channels, causes intense pain due to the release of neuropeptides, such as SP and CGRP [95]. Pre-treatment with BoNT/A dramatically reduces capsaicin-induced mechanical and thermal stimuli, as well as other pain-like behaviors in rodents [58,96]. In humans, the efficacy of BoNT/A on capsaicin-induced hyperalgesia or allodynia has been reported with conflicting results.

Voller *et al.* [90] studied healthy volunteers after intradermal administration of BoNT/A (Botox^®^; 30 U) into forearm *versus* saline. Heat pain threshold and tolerance and neuroselective testing of current pain threshold and tolerance were assessed up to 28 days after treatment, when capsaicin was administered into both forearms. Pain responsiveness and axon reflex flare were evaluated. The results of this study were negative, and BoNT/A showed no effect on pain perception or neurogenic inflammation. Schulte-Mattler *et al.* [88] administered BoNT/A (Dysport^®^; 100 U) *vs.* placebo in defined skin areas of healthy subjects’ forearms and measured the heat and cold pain threshold with QST upon electrical stimulation with a pain-specific surface electrode. Capsaicin-induced flare and allodynia were also measured in the treated skin areas where capsaicin ointment was applied. This study revealed no BoNT/A effect on pain perception or neurogenic inflammation. The negative outcome in these models may be due to insufficient area overlap between BoNT/A and the pain stimulus or an inappropriate dose of BoNT/A [91]. Conversely, other groups have shown that BoNT/A reduces capsaicin-evoked pain and neurogenic vasodilatation in humans [84,86,97]. Tugnoli *et al.* [97] demonstrated that the BoNT/A and capsaicin-treated areas must overlap to demonstrate an inhibition of capsaicin-induced pain sensation, flare area and changes in cutaneous blood flow, similar to Gazerani *et al.* [85,86]. Some aspects of trigeminal sensitization in association with migraine pathophysiology have been mimicked by application of capsaicin in the face and demonstrated that regardless of intramuscular or intradermal injection, BoNT/A is able to reduce pain, neurogenic flare and hyperalgesia induced by capsaicin. In line with the general hypothesis, the effects seen in these studies are most likely due to blockade of substance release (e.g., CGRP, SP, glutamate) by BoNT/A. Differences in capsaicin dose, location and timing are among the potential factors that can yield inconsistent results in different study designs. Recently, Matak *et al.* [98] have demonstrated in their animal models that BoNT/A targeting of TRPV1-expressing neurons might be associated with its selectivity for certain types of pain, which can explain some of the observations in human models.

Ultraviolet B model [99] has also been employed to test the anti-inflammatory and anti-hyperalgesic effect of BoNT/A. Sycha *et al.* [89] applied UVB irradiation. Thermal and mechanical pain and skin blood flow were measured, which remained unaffected in response to BoNT/A (Dysport^®^; 100 U). These negative observations might be due to the fact that the UVB model may be too severe or involve other mechanisms that are not sensitive to the inhibitory action of BoNT/A on substance release [89].

In a series of experiments [72,82,84], it was found that BoNT/A injected into the temporalis muscle significantly reduced intramuscular glutamate-evoked sensitization and vasomotor responses beginning 3 h after injection. The glutamate-induced pain and sensitization model was based on previous studies demonstrating that glutamate plays a role in peripheral sensitization and head pain [100]. Besides, the temporalis muscle is one of the craniofacial muscles injected with BoNT/A in the clinic for migraine prophylaxis [101]. Both the human and animal studies showed a similar line of evidence that pretreatment of temporalis muscle with BoNT/A could inhibit the glutamate-evoked pain and sensitization [72,82]. Bittencourt da Silva *et al.* [82] also presented, for the first time in humans, that BoNT/A decreased pain and cutaneous glutamate release provoked by capsaicin plus mild heat application to the volar forearm of healthy subjects. This dermal microdialysis study was performed in forearm based on the accessibility of the site and ethical issues against the facial region. Results from this study clearly demonstrated that capsaicin evoked glutamate release and that BoNT/A pretreatment dramatically reduced the evoked glutamate release. The outcome is in line with observations from a microdialysis study in rats [102] showing inhibition of glutamate release following a formalin-induced pain model. Hence, it is likely that lowering glutamate concentrations in the tissue can also contribute to the mechanisms of BoNT/A analgesia in humans.

Another potential mechanism involves SV2A, a synaptic vesicle protein isoform with high affinity for BoNT/A that mediates binding and internalization of the neurotoxin into peripheral neurons. Recently, levels of SV2A were investigated in tissues from patients with nerve injury [103]. In addition, the effects of BoNT/A on localization of TRPV1 and functional sensitivity to capsaicin stimuli were determined in cultured human dorsal root ganglion neurons [103]. Results from these studies suggest that differential levels of SV2A protein expression in clinical disorders may identify potential new targets for BoNT/A therapy.

Summing up all of the results reviewed above and referring to the mechanism of action discussed earlier, although an important difference is acknowledged to exist between the peripheral and central sensitization in experimental acute pain *versus* chronic pain conditions, it can be proposed that BoNT/A, following administration in the periphery, interacts with peripheral nociceptive neurons, where it inhibits the release of nociceptive mediators, such as glutamate, SP and CGRP, from peripheral nociceptors. Accordingly, evidence in favor of an effect of BoNT/A on the release of CGRP was also reported by Cernuda-Morollon *et al.* [104], who measured the plasma level of CGRP in 83 patients with chronic migraine after one month from treatment with BoNT/A (Botox^®^; 155–195 U). CGRP levels after BoNT/A treatment were significantly lower as compared to CGRP levels before BoNT/A treatment. Blocking the release of these neurotransmitters inhibits neurogenic inflammation and peripheral sensitization, which potentially blocks the development of central sensitization [91,105,106].

## 5. Use of BoNT/A for Therapeutic Treatment of Headache: Evidence from Clinical Studies

The precise mechanism of BoNT/A as analgesic is still not completely elucidated, and it remains an area of great interest and ongoing research. This notwithstanding, whatever the mechanism of action, the analgesia and low systemic side effects observed in prospective pilot studies have led to the growing interest for the use of BoNT/A for headache therapy. However, systematic large clinical trials using BoNT/A for headache conditions have resulted in a variety of positive and negative findings. In this section, we revise the evidence for the “pros and cons” of the use of BoNT/A as an analgesic for headache conditions. Due to the vastity of the argument involved, we restrict our analysis on the three main types of primary headaches, as summarized in Table 1. A literature search was performed on the PubMed NCBI database, using the word “botulinum” as the search keyword in combination (AND) with either “migraine”, or “headache”, or “tension-type headache”, or “trigeminal”, or “cephalalgia”. Moreover, searches were limited to “clinical trials” as the article type and “humans” as the species. For migraine (Section 5.3), the search was limited to years starting from 2010 to nowadays, because we reviewed only clinical trials after the FDA approval for the use of onabotulinum toxin A for chronic migraine [107].

### 5.1. Tension-Type Headache

TTH is the most common primary headache and one of the most common forms of pain. The pathogenesis of TTH is still not clear, and there are only a few drugs available for the treatment of chronic TTH, the main options being antidepressants. TTH has often been associated with increased pericranial muscle tone [108]. Table 2 summarizes relevant clinical trials where the effects of BoNT/A in the treatment of TTH have been reported.

The use of BoNT/A in TTH was first considered by Relja [109] in an open-label, 12-week prospective study followed, a few years later, by an open-label long-term study (30 patients; 18-month duration), as well as a double-blind, placebo-controlled study (16 patients; eight-week duration) in patients diagnosed for chronic TTH who have been unsatisfactorily treated with standard prophylactic medication, including antidepressants [110]. BoNT/A (Botox^®^; 40–95 U) was injected into the pericranial muscles every three months during the 18 months in an open-label study or only once in the double-blind study. All of the patients injected with BoNT/A showed reduced severity of headache, reduced pericranial muscle tenderness and increased headache-free days during the treatment [110]. In another open-label prospective study [111], 46 patients with a primary clinical diagnosis of chronic TTH coexisting with temporomandibular disorder (TMD) were treated with BoNT/A (Botox^®^; 150 U) injected bilaterally into masseter and temporalis muscles. Subjects were followed on a monthly basis for three months after the injection. The subjects with chronic TTH and TMD symptoms reported a 50% or greater improvement in headache pain. The number of headache-free days also improved post-injection. Smuts *et al.* [112] carried out a double-blind placebo-controlled study, in which 37 patients with chronic TTH received BoNT/A (Botox^®^; 100 U), injected into the temporalis or cervical muscles of the neck. Clinical outcome was measured over a four-month study period using headache diaries and chronic pain index scores. Patients treated with BoNT/A showed an improvement in headache severity over the four-month study period, with the number of headache-free days increased significantly and an improvement in quality of life following BoNT/A injection.

At the same time, well-designed studies failed to show a significant effect of the toxin on improving headache in patients diagnosed with chronic TTH. Schmitt *et al.* [113] conducted a randomized, placebo-controlled study to examine the effect of BoNT/A (Botox^®^; 20 U) injected into frontal and temporal muscles in patients with chronic TTH. During a baseline of four weeks and a post-treatment period of eight weeks, some improvement in affective variables was demonstrated in the botulinum group, but important outcome variables, such as pain intensity, the number of pain-free days and consumption of analgesics, were not statistically different between the groups. However, it should be noted that the dose of BoNT/A used by Schmitt *et al.* [113] was unusually low. A higher dose of BoNT/A (Dysport^®^; 200 U) was used by Rollnik *et al.* [114] in a double-blind, placebo-controlled study with 21 patients diagnosed for TTH. Injections were performed bilaterally into fronto-occipital and temporal muscles. No significant differences between placebo and treatment could be observed with respect to the frequency and duration of headache attacks and quality of life parameters, after 4–12 weeks post-treatment. Gobel *et al.* [115] treated 10 patients each with either BoNT/A (Botox^®^; 20 U) or placebo, but no reduction was found, either in pain intensity, pain-free days or in the use of analgesics. Botulinum toxin was not proven effective in the treatment of chronic tension-type headache also in more significant clinical trials, with a larger number of patients, as reported by Padberg *et al.* [116] and by Schulte-Mattler *et al.* [117].

Silberstein *et al.* [118] conducted a multicenter large trial, enrolling 300 patients, on the efficacy of BoNT/A in chronic TTH. Patients were randomized to receive placebo or different doses of BoNT/A (Botox^®^; 50–150 U), and post-injection follow-up evaluations were on Days 30, 60, 90 and 120. For TTH-free days per month, all groups improved at the Day 60 primary endpoint. There was no statistically-significant difference between placebo and the four BoNT/A groups, but a significant difference, favoring placebo *vs.* BoNT/A 150 U, was observed. At Day 90, significantly more patients reported a 50% decrease in headache days in several BoNT/A groups, suggesting that a longer period of evaluation may be needed to see a treatment effect.

Contrasting results from prophylactic treatment of TTH with BoNT/A may be related to a mix of not well-controlled and standardized conditions, such as the injection protocol, the BoNT/A dosage range, the patient population and its numerical consistency or the use of concomitant prophylactic headache medication with other drugs.

**Table 2:** Clinical trials with botulinum neurotoxin A (BoNT/A) in patients diagnosed for TTH.

Authors	Study ^(1)^	Patients ^(2)^	BoNT/A ^(3)^	Injected Muscles ^(4)^	Outcomes ^(5)^	Ref.
Gobel *et al.*, 1999	DP/6 w	10 (+10)	B 20 U	frontal, auricular, splenium	−	[115]
Smuts *et al.*, 1999	DP/4 m	37 (+15)	B 100 U	temporalis, cervical	+	[112]
Rollnik *et al.*, 2000	DP/4–12 w	11 (+10)	D 200 U	fronto-occipital, temporal	−	[114]
Schmitt *et al.*, 2001	DP/4–8 w	30 (+29)	B 20 U	frontal, temporalis	−	[113]
Freund and Schantz, 2002	PO/3 m	46	B 150 U	masseter, temporalis	+	[111]
Padberg *et al.*, 2004	DP/12 w	19 (+21)	B 100 U	multiple pericranial	−	[116]
Relja and Telarovic, 2004	PO/18 m	30	B 45–90 U	multiple pericranial	+	[110]
Relja and Telarovic, 2004	DP/8 w	8 (+8)	B 45–90 U	multiple pericranial	+	[110]
Schulte-Mattler *et al.*, 2004	DP/12 w	53 (+54)	D 500 U	multiple pericranial	−	[117]
Silberstein *et al.*, 2006	DP/4 m	250 (+50)	B 50–150 U	multiple pericranial	+/−	[118]

(1) PO = prospective open-label; DP = double-blind placebo-controlled; w = weeks; m = months; duration time indicates total time of study including post-treatment period. (2) In DP studies, patients were randomized into two groups, and (+*n*) indicates the number of patients who received placebo injections (saline). (3) Maximal doses of injected BoNT/A; B = Botox^®^; D = Dysport^®^. (4) Depending on the protocol, injections may be single or repeated at regular intervals, mono- or bi-lateral and single or multiple sites. (5) Positive (+) outcomes indicate any statistical improvements in important outcome variables, such as pain intensity, the number of pain-free days, consumption of analgesics, frequency of headache attacks and quality of life parameters. Negative (−) outcomes indicate no statistical differences between the BoNT/A and placebo groups.

Recently, Jackson *et al.* [119] performed a meta-analysis on randomized controlled trials on the use of BoNT/A in association with chronic TTH and concluded that the majority of randomized, double-blind and placebo-controlled trials do not confirm the assumption that BoNT/A could be a prophylactic efficacious treatment of TTH disorders. In summary, the weight of the evidence “pro and con” for the use of BoNT/A in chronic TTH actually is unbalanced towards “con”, and further studies are needed to completely clarify this issue.

The lack of a proven efficacy of BoNT/A on TTH has two faces. On the one side, the lack of effect of its myorelaxant action confirms the scarce role of muscle hyperactivity in TTH (in spite of its name); on the other side, it suggests a mechanism of pain for which the toxin does not work, in spite of some similarities and the not so infrequent copresence of TTH and migraine in the same patient.

### 5.2. Trigeminal Autonomic Cephalalgias and Trigeminal and Occipital Neuralgias

Relevant clinical trials for the treatment of TACs and trigeminal and occipital neuralgias with BoNT/A are summarized in Table 3.

Botulinum toxin type A has recently been studied as a new preventive treatment for patients with TACs, mainly CH, with limited success. Only one open-label single-center study evaluated the efficacy and tolerability of BoNT/A in the treatment of CH [120]. Twelve male patients with episodic (*n* = 3) or chronic (*n* = 9) CH were treated with a cumulative dose of BoNT/A (Botox^®^; 50 U) into the ipsilateral pericranial muscles. The effect of BoNT/A was limited. One patient with chronic CH experienced a total cessation of attacks, and in two patients, attack intensity and frequency were improved. In another patient with chronic CH, typical attacks were not influenced, but an ipsilateral continuous occipital headache was improved significantly. Patients with episodic CH did not benefit from BoNT/A treatment. Although these findings provide evidence that BoNT/A may be beneficial as a therapy for patients with chronic CH, as of yet, no multicenter, randomized, controlled studies for CH have been published to confirm the results seen in this open-label study.

Instead, several reports concern the use of BoNT/A for the treatment of trigeminal (TN) and occipital neuralgia (ON). TN is a severe chronic pain syndrome characterized by an excruciating, brief electric shock-like paroxysmal pain in one or more branches of the trigeminal nerve. It can occur either spontaneously or upon gentle tactile stimulation of a trigger zone on the face or in the oral cavity [121,122]. Till now, there are no specific drugs for TH, and the pharmacotherapy of TN includes the use of antiepileptic drugs, like carbamazepine, baclofen, lamotrigine, gabapentin or sodium valproate, among others [121]. In the past few years, several reports on the successful use of BoNT/A in patients with TN seem to open a new way to contrast this refractory chronic pain.

In an open-label pilot study, Borodic and Acquadro [123] injected BoNT/A (Botox^®^; 30–50 U) as multifocal injections over the dermatome where pain was experienced in 11 patients diagnosed for TN, and eight patients responded positively to treatment. Piovesan *et al.* [124] observed a nearly complete pain relief in 13 patients after subdermal injections of BoNT/A (unspecified; 3.22 U/cm^2^) directly into the affected facial regions, among the branches of the trigeminal nerve, for 10 days. Turk *et al.* [125] injected BoNT/A (Botox^®^; 100 U) into the region of the zygomatic arch of eight patients and found that it was effective at treating TN. Zuniga *et al.* [126] treated 12 patients of TN with BoNT/A (Botox^®^; 20–50 U) injected subcutaneously in divided doses at various trigger zones along the involved branch of the trigeminal nerve. Patients were evaluated at weekly interval for eight weeks, and 10 patients benefited from BoNT/A and remained pain-free for an average period of 60 days. Beneficial effects of BoNT/A injections into the trigger zone of TN were observed also in another pilot [127] and in two case report [128,129] studies.

After positive outcomes from these prospective studies, double-blind and placebo-controlled trials were conducted. In a 12-week follow-up randomized, double-blind, placebo-controlled trial, Wu *et al.* [130] injected BoNT/A (Lanzhou Biological Products Institute; 75 U) intradermally and/or submucosally into trigger zones of 22 patients, while 20 patients received placebo, diagnosed for TN. BoNT/A significantly reduced pain intensity at Week 2 and pain attack frequency at Week 1. The efficacy was maintained throughout the course of the study: BoNT/A-treated patients reported that pain had improved by the end of the study. This positive result was further confirmed by Zhang *et al.* [131]. In another trial, Shehata *et al.* [132] treated 20 patients with intractable TN. Patients were randomized in a double-blind way, and each patient received either BoNT/A (Botox^®^; 100 U) or placebo, and it was found that pain reduction at the 12-week endpoint was significant in the BoNT/A group. Beneficial effects of BoNT/A were also reported by Xia *et al.* [133], who injected, in the painful area, 87 patients with one-branch classical TN, and it was found that BoNT/A treatment can significantly relieve the pain; moreover, a reduction of anxiety, depression and sleep disorders together with an increase of the quality of life were observed.

**Table 3:** Clinical Trials with BoNT/A in patients diagnosed for CH, trigeminal (TN) or occipital neuralgia (ON).

Authors	Study ^(1)^	Patients ^(2)^	BoNT/A ^(3)^	Injection Sites ^(4)^	Outcomes ^(5^^)^	Ref.
*Cluster Headache*						
Sostak *et al.*, 2007	PO/3–10 m	12	B 50 U	pericranial muscles	+/−	[120]
*Trigeminal Neuralgia*						
Borodic and Acquadro, 2002	PO/4 m	11	B 30–50 U	dermatome with pain	+	[123]
Allam *et al.*, 2005	CR/3 m	1	B 16 U	hemifacial region	+	[128]
Piovesan *et al.*, 2005	PO/2 w	13	n.a.	subdermal facial region	+	[124]
Turk *et al.*, 2005	PO/n.a.	8	B 100 U	zygomatic arch	+	[125]
Zuniga *et al.*, 2008	PO/8 w	12	B 20–50 U	subcutaneous trigger zone	+	[126]
Ngeow *et al.*, 2010	CR/5 m	1	B 100 U	nasal and mental trigger zone	+	[129]
Bohluli *et al.*, 2011	PO/6 m	15	B 50 U	trigger zone	+	[127]
Wu *et al.*, 2012	DP/12 w	22 (+20)	L 75 U	intradermal skin or oral mucosa	+	[130]
Sheata *et al.*, 2013	DP/12 w	10 (+10)	B 100 U	subcutaneous at trigger zone	+	[132]
Zhang *et al.*, 2014	DP/8 w	56 (+28)	L 25–75 U	intradermal skin or oral mucosa	+	[131]
Xia *et al.*, 2015	DP/8 w	47 (+40)	L 50 U	facial pain area	+	[133]
*Occipital Neuralgia*						
Volcy *et al.*, 2006	CR/10 m	1	n.a. 40.5 U	masseter and zygomatic muscles	+	[134]
Kapural *et al.*, 2007	PO/4 w	6	B 50–100 U	greater occipital nerve	+	[135]
Taylor *et al.*, 2008	PO/12 w	6	B 50 U	greater occipital nerve	+/−	[136]

(1) CR = case report; PO = prospective open-label; DP = double-blind placebo-controlled; w = weeks; m = months; duration time indicates total time of study including post-treatment period. (2) In DP studies, the patients were randomized into two groups, and (+*n*) indicates number of patients who received placebo injections (saline). (3) Maximal doses of total injected BoNT/A; B = Botox^®^; L = Lanzhou Biological Products Institute, China; n.a. = not available. (4) Depending on the protocol, injections may be single or repeated at regular intervals, mono- or bi-lateral and single or multiple sites. (5) Positive (+) outcomes indicate any statistical improvements in important outcome variables, such as pain intensity, the number of pain-free days, consumption of analgesics, frequency of headache attacks and quality of life parameters. Negative (−) outcomes indicate no statistical differences between the treated and placebo groups.

From these studies, it can be reasonably concluded that BoNT/A may provide a clinically-significant benefit for the treatment of adult TN patients. However, well-designed randomized, controlled, double-blinded trials with a larger number of patients are still lacking, and future adequately-powered studies are needed.

ON was another neuralgia where a positive effect of BoNT/A was reported; one case report and two prospective studies are available up to now [134,135,136]. ON is defined as a paroxysmal shooting or stabbing pain in the dermatomes of the nervus occipitalis major and/or nervus occipitalis minor. The pain originates in the suboccipital region and radiates over the vertex. Kapural *et al.* [135] describe a series of six patients with severe ON who received conservative and interventional therapies without significant relief. This group then underwent occipital nerve blocks using the BoNT/A (Botox^®^; either 50 U or 100 U if bilateral). A significant decrease in pain was observed at four weeks follow-up in five out of six patients following BoNT/A occipital nerve block. Taylor *et al.* [136] injected BoNT/A (unspecified; 50 U) into regions traversed by the greater and lesser occipital nerve in six subjects diagnosed with ON. The sharp/shooting type of pain showed improvement during most of the trial period. Furthermore, the headache-specific quality of life exhibited some significant improvement by six weeks that continued through Week 12. However, no significant reduction in pain medication usage was demonstrated. Results from these pilot studies suggest that further large placebo-controlled trials are warranted before indicating BoNT/A as an alternative therapy against ON.

### 5.3. Migraine

According to different studies, 2%–15% of the world’s population suffers from migraine, which is characterized by frequently severe headaches, often accompanied with nausea, vomiting and increased sensitivity to sound and light. Attacks may widely vary in frequency. If they occur on 15 or more days per month (with the feature of migraine on at least eight days per month), the disorder is called “chronic migraine” (CM) based on the International Classification of Headache Disorders (ICHD-3; [26]), otherwise, it is an “episodic migraine” (EM). The former is obviously more uncomfortable, and therefore, its treatment is a major challenge.

The commonly-used prophylaxis agents for migraine include adrenergic blockers, calcium channel blockers, tricyclic antidepressants and anticonvulsants. Due to the limited efficacy of the currently available therapies and also the undesirable safety profile of the majority, many efforts have been done to find new drugs for migraine treatment, and this has led to examining BoNT/A.

Systematic double-blind, placebo-controlled randomized trials using BoNT/A (mainly Botox^®^) as a therapeutic drug for migraine have resulted in a mix of positive and negative findings (see Table 1 in [137]). Participants in these trials were patients suffering from either EM (less than 15 attacks per month; 11 trials) or CM (more than 15 attacks per month; 10 trials). Out of 11 clinical trials in patients suffering from EM, only three showed the efficacy of BoNT/A in reducing migraine symptoms. Thus, up to date, based on the available data, BoNT/A has not been convincingly shown to be effective in the prevention of EM [138].

In CM prophylaxis, available randomized, double-blind, placebo-controlled trials suggest that BoNT/A is effective at improving headache symptoms and quality of life. In fact, out of 10 clinical trials in patients suffering from CM, only two reported negative results (see Table 1 in [137]). Based on these available data, on 15 October 2010, the new indication for the use of BoNT/A for the prophylaxis of headaches in adults with CM was approved by the U.S. agency, FDA [107], followed by approval from European and National agencies in recent years. Approval came from evidence presented to the agencies from two large studies conducted in North America and Europe entitled Phase III Research Evaluating Migraine Prophylaxis Therapy (PREEMPT 1 and 2; 1384 patients enrolled across both trials), funded by Allergan Inc., showing a reduction in the frequency of migraine attacks for migraine sufferers undergoing the Botox^®^ treatment [139,140]. The total dose of injected BoNT/A ranged from 155 U–195 U, administered to 31 sites in seven head and neck muscles every 12 weeks for two cycles with the first injection at the starting day. Both PREEMPT trials had two endpoints: the primary endpoint was the change in frequency of headache episodes at Week 24 compared to the baseline, while the secondary endpoint was the change in frequency of headache days at Week 24 compared to the baseline. PREEMPT 1 showed no significant improvement in the frequency of headache episodes, but significant reduction in the frequency of headache days, while PREEMPT 2 showed significance both in the reduction of the frequency of headache days and the frequency of headache episodes. Pooled together, the combined results of PREEMPT 1 and 2 were significant, but the therapeutic gain over placebo was only an 11% significant reduction in headache days after six months [141]. Although it is still open to debate whether such low efficacy would be effective in refractory CM, significant improvements in treated patients *vs*. placebo groups were obtained in other variables, such as the frequency of severe headache days, the cumulative hours of headache per day and the proportion of patients with severe disability. In a subgroup of the PREEMPT study, Silberstein *et al.* [142] analyzed patients who had medication overuse (MO) together with chronic migraine (MO + CM; 65,3% of 1384 patients). At 24 weeks, MO + CM patients demonstrated significant reduction of headache days compared to placebo, and triptan intake was significantly reduced in MO + CM patients after BoNT/A treatment. The authors concluded that BoNT/A treatment is effective not only in CM patients without MO, but also with MO.

Continued development of BoNT/A for CM focused on longer term studies to establish the long-term efficacy and safety of BoNT/A. For example, PREEMPT studies continued, and data up to 56 weeks have been published [143]. In this last trial, the two previous phase III studies (PREEMPT 1 and 2) of the 24-week double-blind placebo-controlled phase with two cycles of BoNT/A, at the starting day and 12 weeks, were followed by a 32-week open-label phase with three more cycles of BoNT/A at 24, 36 and 48 weeks. Of 1384 original PREEMPT patients, 1005 continued and received all five cycles of treatment. At the end of the study, 513 patients received five cycles of BoNT/A, and 492 received the first two cycles of placebo, then three cycles of BONT/A. At Week 56, patients receiving five cycles of BoNT/A showed better improvements in the frequency of headache days than patients receiving two cycles of placebo plus three cycles of BoNT/A, suggesting that patients treated earlier had better outcomes. These findings demonstrate the continued need and cumulative benefit over time with continued prophylaxis, an important and clinically-pragmatic observation for clinicians and patients.

In the last two years, longer term studies have been initiated and concluded or are currently ongoing. By using medical records data, the phase IV study entitled CLARITY [144] evaluated the durability of benefit in 33 patients that received 7–9 BoNT/A (Botox^®^; 155–195 U) cycles for treatment of CM. The results obtained warrant investigation in a larger study to better understand the durability of BoNT/A benefit for CM in clinical practice. A 108-week phase IV clinical trial, entitled Chronic migraine Onabotulinu M toxin A Prolonged Efficacy open Label COMPEL [145,146], intends to provide additional information about and the long-term response of the CM population to BoNT/A (Botox^®^; 155 U) treatment. In a very recent study [147], 132 patients with CM were injected with BoNT/A (Botox^®^; 100–200 U or 155–195 U) quarterly during the first year, and the fifth visit was delayed to explore the need for further injections. A total of 108 patients (81.8%) showed a response during the first year. Among those 108 patients with treatment longer than one year, injections were stopped in 10 due to a lack of response and in four due to the disappearance of attacks. In responders, after an average of two years of treatment, consumption of any acute medication was halved. These results confirm the long-term response to BoNT/A in CM patients.

At the end of this review, we want to underline again that both central and peripheral mechanisms are responsible for CM. We should recall the attention of the reader to the fact that also the vascular structure, that is the nociceptive fibers surrounding the arteries and possibly veins, is believed to be a site of peripheral mechanisms. The involved arteries are not only intracranial, but also extracranial (scalp). In this respect, several experimental and clinical data are available (reviewed in [29]). This has important implications on the mode and sites of injection of BoNT/A. In the practice of CM treatment, BoNT/A is prevalently injected inside pericranial muscles located either in the face (procerus, corrugator, frontalis) or in the temporal side of the head (temporalis) or in the neck (occipitalis, splenius/paraspinalis, upper part of trapezius) [148,149,150,151]. This has been done since the first clinical trials without support of pathophysiological data: in fact, there is no evidence for a role of pericranial muscles on the pathogenesis of CM, a role suggested instead for TTH, which, however, does not seem to substantially benefit from intramuscular BoNT/A. On the basis of the results of the study by Del Fiacco *et al.* [61], who found increased TRPV1 in the superficial temporal artery of patients with chronic migraine, and of the data indicating a role of neurovascular scalp structures in migraine [29], Silberstein [152] suggests to inject BoNT/A mainly in proximity of scalp arteries instead of muscles. The results of the report from Luvisetto *et al.* [58] support this suggestion, indicating a direct invasion of nociceptive fibers by the toxin. BoNT/A injected into the scalp structures outside the muscles, besides an action through the extracranial meningeal collaterals nociceptors, as suggested by Burstein *et al.* [65], can act by reaching to the easily-accessible TRP-positive perivascular nerve fibers. Moreover, intramuscular injection probably favors the uptake of the toxin by the rich network of motor nerve endings, reducing the uptake by sensory endings; therefore, extramuscular injection could lead to a reduction of the necessary toxin doses. Therefore, at least one clinical trial seems reasonable to clarify this claim. The comparison of the results obtained from the two different injection sites can be also useful for a better identification of the pathophysiological mechanisms.

## 6. Conclusions

There is much basic science evidence for an analgesic effect of BoNT/A, and clinical trials confirmed the efficacy, safety and tolerability of the toxin in the prophylactic treatment of certain headaches in adults, such as chronic migraine and also trigeminal neuralgia, but not for tension-type headache. In summary, our current notion is that BoNT/A exerts a prophylactic effect through a dual mechanism, consequent to inhibition of SNARE-mediated synaptic vesicle trafficking, by inhibiting the peripheral release of neurotransmitter and inflammatory neuropeptide-containing vesicles (e.g., glutamate, CGRP, substance P) and by interfering with the cell surface expression of relevant peripheral receptors and ion channels (e.g., TRPV1, TRPA1). In patients, BoNT/A is injected into muscles of the craniofacial-cervical region innervated by trigeminal nerve branches. Accordingly, peripheral sensitization is disrupted, and central sensitization is indirectly blocked, resulting in an antinociceptive response in the sensitized trigeminal nerve and cervical afferents. However, before translating experimental results into guidelines for possible use in the clinical setting, it is worth considering all potential important differences that may exist in both peripheral and central sensitization between experimental acute pain and chronic pain in humans.
